# Case report: Pulmonary synovial sarcoma in a long-term survivor of childhood Hodgkin lymphoma

**DOI:** 10.3389/fonc.2023.1096160

**Published:** 2023-02-24

**Authors:** Konstantin Shilo, Peter J. Kneuertz, David Liebner, Wei Chen

**Affiliations:** ^1^ Department of Pathology, The Ohio State University Wexner Medical Center, Columbus, OH, United States; ^2^ Division of Thoracic Surgery, The Ohio State University Wexner Medical Center, Columbus, OH, United States; ^3^ Division of Medical Oncology, The Ohio State University Wexner Medical Center, Columbus, OH, United States

**Keywords:** synovial sarcoma, SS18 (SYT) rearrangement, SS18-SSX1 fusion, second malignancy, childhood Hodgkin lymphoma, survivor

## Abstract

Solid organ malignancies have been reported in survivors of Hodgkin lymphoma treated with chemoradiation; however, to the best of our knowledge no cases of pulmonary synovial sarcoma have been documented in the literature in this cohort. We herein provide a detailed description of synovial sarcoma occurring in the lung of a long-term survivor of childhood Hodgkin lymphoma. A 29-year-old female never smoker with past medical history of Hodgkin lymphoma diagnosed at the age of 7 years and treated with chemotherapy and radiation therapy was admitted for management of pneumothorax. Wedge lung resection of an ulcerated subpleural nodule revealed a malignant spindle cell tumor that based on light microscopic and immunohistochemical features was classified as monophasic synovial sarcoma. The diagnosis was further confirmed by identification of SS18 (SYT) rearrangement by fluorescence *in situ* hybridization and SS18-SSX1 gene fusion by RNA sequencing. The case documents a rare occurrence of synovial sarcoma in a long-term survivor of childhood Hodgkin lymphoma. While comprising a typical genetic profile for synovial sarcoma, the tumor had unusual histological features such as cystic and low-grade morphology. The case suggests that synovial sarcoma falls within an expanding spectrum of secondary malignancies following prior treatment of Hodgkin lymphoma.

## Introduction

As more childhood Hodgkin lymphoma (HL) patients achieve disease free adulthood, they face increased risk of second malignancies associated with side effects of radiation and chemotherapy (1–3). Secondary solid organ malignancies have been reported in this cohort; however, to the best of our knowledge no cases of pleuropulmonary synovial sarcoma (SS) have been documented in the literature.

SS is a rare soft tissue sarcoma that also affects visceral organs including lung (4). It is associated with a specific chromosomal translocation, which most commonly fuses SS18 (SYT) in chromosome 18 and SSX1 or SSX2 in chromosome X (5). The tumor tends to occur in young adults and shows no sex predilection. The presenting symptoms are nonspecific mostly related to the site of origin. Rare occasions of SS presenting with pneumothorax have been reported (6–9).

We herein provide a detailed description of pulmonary SS in a long-term survivor of childhood Hodgkin lymphoma and highlight some unusual clinicopathological features.

## Case presentation

**Figure 1:**
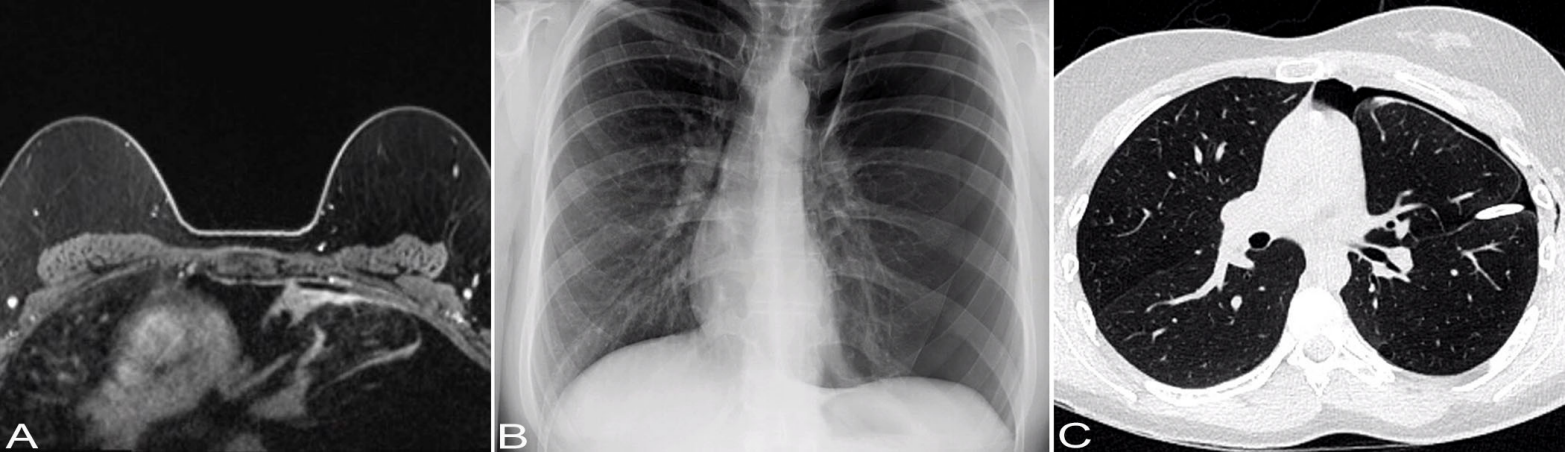
Imaging findings. **(A)** Axial non-contrast T1-weighted breast MRI image shows no evidence of malignancy. There is a mediastinal shift with the heart shifted to the right and a lack of lung markings in the upper left lung, suggesting the possibility of bulla in the upper left lung. **(B)** Chest X-ray shows a left-sided pneumothorax with cardiac and mediastinal shift rightward suggesting underlying tension component. **(C)** Chest CT shows a small left anterior pneumothorax. There is a left-sided pleural drainage catheter with its tip along the left major fissure. There are subtle patchy consolidative opacities in the subpleural aspects of the left upper lobe.

**Figure 2:**
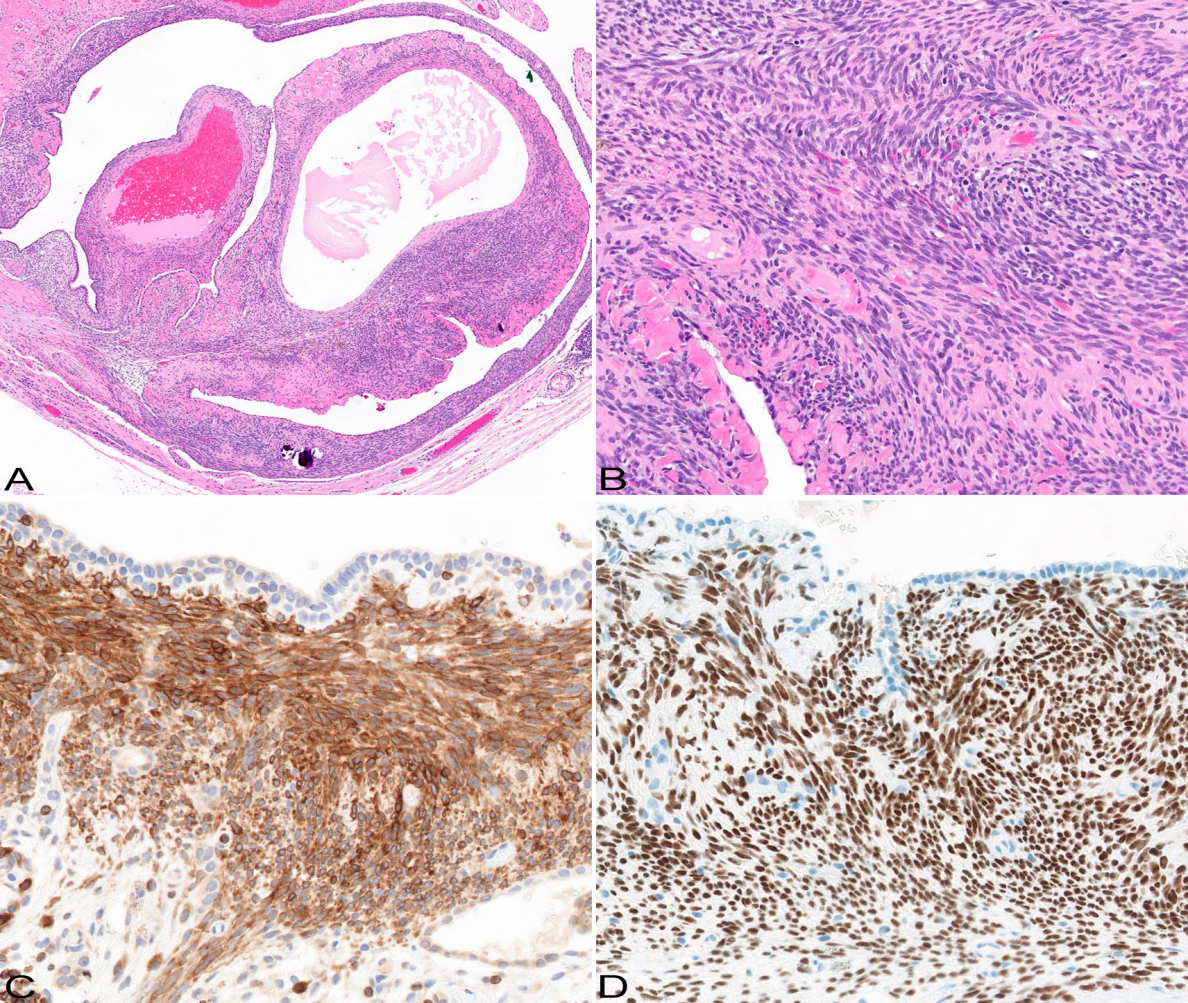
Pathological features. The tumor is represented by cystic and solid components **(A)**; HE original magnification x5. The solid component is composed of spindle cells forming fascicles **(B)**; there is variable background collagen deposition; HE original magnification x20. The tumor cells are immunoreactive with bcl2 **(C)** and SS18-SSX **(D)** antibodies; immunohistochemistry, original magnification x20 and x20.

A 29-year-old female never smoker was admitted for inpatient management of a left sided pneumothorax (PTX) that was discovered on a screening breast magnetic resonance imaging (MRI). The patient’s past medical history was significant for HL diagnosed at the age of 7 years. She was treated with DBVE chemotherapy which included Doxorubicin (D), Bleomycin (B), Vincristine (V) and Etoposide (E), and involved field radiation therapy (IFRT) to cervical spine, supraclavicular area, and middle and lower mediastinum (2,550 cGy administered in 17 fractions of 150 cGy each) according to a pediatric oncology group study (POG-9426) (10). Her past medical history was also notable for hypothyroidism, migraine, and polycystic ovarian syndrome. Breast MRI showed no evidence of malignancy but was significant for a mediastinal shift with the heart shifted to the right (**Figure 1A**). Chest X-ray (CXR) confirmed left PTX with slight cardiac and mediastinal shift rightward suggesting underlying tension component (**Figure 1B**). As a chest tube placement did not produce complete resolution of the PTX (**Figure 1C**) and she endorsed a persistent air leak, a video assisted thoracoscopic surgery (VATS) was performed for pleurodesis. The intraoperative findings included severe pleural adhesions and a dense lung parenchyma. A focal umbilication and surface disruption of the visceral pleural surface of left upper lobe was detected, which was thought to be site of the air leak. On further palpation a subpleural ulcerated nodule was discovered. A VATS lung wedge resection of the nodule was performed with clear margins. There was no evidence of pleural seeding at thoracoscopy. Gross examination showed a 1.3 x 0.8 x 0.4 cm cystic nodular lesion abutting visceral pleura. On light microscopic examination, the lesion was represented by small cysts associated with spindle cell proliferation of variable cellularity (**Figure 2A**). The cystic spaces were partially lined by entrapped nonneoplastic pneumocytes and partially lined by respiratory epithelium. The spindle cells were arranged in fascicles (**Figure 2B**). There was variable amount of collagen, areas of fibrous scarring and microscopic calcifications. There were rare apoptotic bodies and rare mitotic figures (1 per 10 high power fields). The tumor cells were immunoreactive with CD56, BCL2 and SS18-SSX (clone E9X9V, Cell Signaling Technology, Danvers, MA) (**Figures 2C, D**). Rare tumor cells were weakly positive for Keratin AE1/AE3, Synaptophysin and Estrogen Receptor (ER). Immunostains for Calretinin, WT1, CD68, PLAP, Inhibin, Smooth Muscle Actin, Desmin, Myogenin, CD34, STAT6, SOX10, HMB45, CD117a, Progesterone Receptor (PR), CD10, PAX8, CK7, CK20, CDX2, p40 and TTF1 were negative. Ki67 proliferation rate was approximately 1-3%. The tumor showed SS18 rearrangement by fluorescence *in situ* hybridization (FISH). Gene rearrangement analysis from RNA sequencing (Tempus xT, targeted panel of 648 genes) reveled SS18-SSX1 gene fusion, with tumor mutation burden of 0 m/Mb, and stable microsatellite instability status. Based on the morphological and immunohistochemical and genetic findings, the tumor was classified as monophasic SS. As per a multidisciplinary conference review, no adjuvant therapy was recommended at the time. Two months following initial diagnosis, restaging computed tomography (CT) and fused positron emission tomography/computed tomography (PET/CT) showed no residual disease in the chest and no distant metastatic disease. No evidence of recurrence was documented at 5-month follow-up chest CT. The patient was well without evidence of disease at 1 year following the initial diagnosis. A strategy of close radiographic surveillance with serial chest CT (or PET/CT for equivocal findings) for at least 10 years was recommended.

## Discussion

HL survivors demonstrate increased risk for developing radiation-related breast cancer, lung cancer and colorectal cancer and their risk appears to be associated with the site of radiation (3). Radiation associated sarcomas with nonrecurrent complex genetic alterations following HL treatment are well documented in the literature (11, 12). They characteristically develop after a latency period of one to two decades within radiotherapy fields and majority represent malignant fibrous histiocytoma and osteosarcoma (11, 12). Sarcomas carrying specific translocations such as Ewing sarcoma are relatively rare (13–16). To the best of our knowledge only a single case report of an infraclavicular SS arising at the site of prior radiation for HL has been documented in the literature (17). In contrast to our patient, a patient reported by Van De Rijn at al developed SS in infraclavicular soft tissue, showed locally aggressive behavior with involvement of brachial plexus and based on the microscopic description had no cystic morphology.

Our patient was treated with IFRT and DBVE chemotherapy for HL according to the POG-9426 protocol and developed SS after 22 years. While it is not yet entirely certain if the risk of the development of second malignancy is associated with therapy or with genetic background of patients or both, the occurrence of SS at the site of prior radiation following latency period of about 2 decades is consistent with a notion of a possible causative association between prior chemoradiation and the development of second malignancy in our case. The lung is not intended target during mantle radiotherapy for HL and radiation dose across the lung can vary several folds (18). Nevertheless, the medial aspects of the lung including visceral and parietal pleura overlap with the mediastinal component of the radiation fields and therefore, the visceral pleura of the left upper lobe, the site of the tumor in our patient, is at the increased risk of radiation induced malignancy. Some sarcomas, such as rhabdomyosarcoma, malignant peripheral nerve sheath tumor and liposarcoma, are known to be associated with germline alterations, while others, such as Ewing sarcoma, show no known hereditary associations (19). Possibly due to its rarity, the evidences for hereditary predisposition for SS are currently lacking.

The majority of SS are highly proliferative tumor that fall within FNCLCC grade 2 and 3, however, a low percentage (up to 10%) of SS show low mitotic rate (less than 10 per 10 high power fields), as the tumor in this case (20–22). While the overall histological and genetic features of the tumor in this report fall within the spectrum of that typically seen in SS, it also showed some unusual findings including the small size (1.3 cm), cystic morphology, and low mitotic/proliferation rate. The cystic morphology of the tumor was likely responsible for the clinical presentation as PTX.

Cystic primary pulmonary SS as a small subset have been recognized to present with PTX and manifest pathologically as cystic or bullous lesions with only subtle evidence of a malignant proliferation (6, 8). The original publication reported 4 patients (ages 20 to 29) all with a monophasic variant of SS and emphasized that since these tumors manifest primarily as cystic lesions presenting as PTX, they can be confused with benign cystic lung disease and therefore in the settings of PTX a thorough examination of all resected tissue with increased cellularity should be carried out (6). As of date, 7 cases of pleuropulmonary SS with cystic morphology have been reported (6–9). As in our case, all 7 of 7 cases were monophasic SS, 3 of 7 cases were small less than 1.5 cm lesions, 2 of 7 had microscopic calcifications and 3 of 3 tested cases demonstrated the gene fusion product SS18-SSX1, similar to that in our case. None of these 7 cases specifically reported history of prior malignancy including HL.

Since the presence of specific chromosomal translocation is a requirement for the diagnosis of SS, it is the most decisive feature to help to exclude other lesions. However, at the initial work-up or if molecular testing is not available, lymphangioleiomyomatosis (LAM) and pulmonary involvement by endometriosis have to be excluded, especially in young adult female patients. PTX at presentation and the presence of spindle cell proliferation associated with parenchymal cysts are the findings that are shared between SS and LAM (23–25). However, LAM represents a diffuse cystic lung disease associated with proliferation of so called perivascular epithelioid cells that characteristically show aberrant expression of melanocytic markers (e.g. HMB45, Melan A) and therefore their use will help to exclude the diagnosis of LAM at immunohistochemical level. While uncommon, the pulmonary endometriomas can be seen in the setting of endometriosis and present with catamenial PTX. The overlapping features between endometriosis and SS include the presence of spindle cell stroma showing CD56 expression (26). However, in endometriosis expression of other markers of Müllerian origin such as ER, PR and PAX8 will help to distinguish it from SS.

## Conclusion

This report documents a rare case of SS arising in a long-term survivor of childhood HL and suggests its possible association with prior therapy. The tumor’s cystic morphology is likely responsible for unusual clinical presentation as PTX.

## Data availability statement

The original contributions presented in the study are included in the article/supplementary material. Further inquiries can be directed to the corresponding author.

## Ethics statement

Written informed consent was obtained from the individual for the publication of any potentially identifiable images or data included in this article.​

## Author contributions

PK and DL participated in data collection. WC and KS participated in the conception of the study. KS wrote the manuscript text. PK, DL, WC, and KS edited the manuscript. All authors contributed to the article and approved the submitted version.
